# Elevated N-telopeptide as a potential diagnostic marker for bone metastasis in lung cancer: A meta-analysis

**DOI:** 10.1371/journal.pone.0187860

**Published:** 2017-11-28

**Authors:** Boxuan Liu, Yun Zhao, Jingyan Yuan, Lizhong Zeng, Ruiying Sun, Xia Meng, Shuanying Yang

**Affiliations:** 1 Department of Respiratory Medicine, the Second Affiliated Hospital, Xi'an Jiaotong University, Xi'an, Shaanxi, P.R., China; 2 Department of Cardiovascular Medicine, the First Affiliated Hospital, Xi'an Jiaotong University, Xi'an, Shaanxi, P.R., China; Virginia Commonwealth University, UNITED STATES

## Abstract

**Background:**

Growing evidence indicates that the cross-linked N-telopeptide of type I collagen (NTx) is likely to be involved in the development of bone metastasis among lung cancer patients. We perform a meta-analysis to disclose the correlation between bone metastasis and NTx and also to evaluate its value in diagnosis of bone metastasis (BM) in lung cancer.

**Method:**

Electronic databases were searched and calculated the weighted mean difference (WMD) with 95% confidence interval (CI) to assess the expression difference of NTx between BM+ and BM- lung cancer patients. Moreover, we conducted a sensitivity and specificity test and drew a summary receiver operating characteristic curve (SROC) to assess the diagnostic value of NTx in discerning bone metastasis.

**Results:**

A total of eleven studies with 1108 individuals were included in this analysis. The results showed an increased NTx was correlated with the incidence of lung cancer (P < 0.001). The overall sensitivity and specificity of serum NTx (sNTx) for discerning bone metastasis was 0.74 (95% CI = 0.67 to 0.79) and 0.85 (95% CI = 0.80 to 0.89), respectively. As for urine NTx (uNTx) the pooled sensitivity and specificity was 0.77(95% CI = 0.67 to 0.86) and 0.81(95% CI = 0.76 to 0.86). The area under the SROC curve was 0.8889(SE = 0.0255) and 0.8655(SE = 0.0254) for sNTx and uNTx respectively.

**Conclusions:**

The elevation of NTx in lung cancer was positively related with the development and progression of bone metastasis. A higher specificity over sensitivity of NTx suggested that it is a more accurate biomarker to distinguish patients without bone metastasis. Regarding SROC curve, sNTx may be a better choice.

## Background

Lung cancer has been the most common malignancy for several decades with an estimated 1.8million new cases annually and resulted in one cancer death in five (1.59 million deaths,19.4% of total)[1]. A majority of lung cancer patients are in advanced stage at the time of diagnosis and bone is tended to be the most common affected site with a rough rate of 30% to 60%[2]. The occurrence of skeletal-related events (SREs) such as pathologic fracture and hypercalcemia[3]followed by bone metastasis (BM) will not only result in a poor quality of life but also can be costly. Nowadays, the diagnosis of bone metastasis in cancer patients relies predominantly on imaging techniques, such as bone scintigraphy which often has a high sensitivity but lacks specificity[4], computerized tomography (CT), magnetic resonance imaging (MRI) or18F-fluorodeoxyglucose positron emission tomography (F-18 FDG-PET). Although these techniques have been proved to be useful diagnostic tools, their limitations on early diagnosis or time-to-time monitoring cannot be ignored. Thus, these weaknesses of current methodology lead to a need for establishing supplementary diagnostic tools.

The cross-linked N-telopeptide of type I collagen (NTx) is one of the degradation products of type I collagen which accounts for 90% of organic chemical components of bone[5]. It is highly bone specific and its levels are often increased when bone metastasis occurs[6]. Previous study conducted by Zhang[7] has demonstrated that NTx may have a value in the diagnosis of bone metastasis, however, this study lacks sufficient data to evaluate the overall sensitivity and specificity for NTx, moreover the patients included in the study are all Chinese and with various solid tumors (breast and prostate cancer) which cannot be fully applied in lung cancer patients. Therefore, we comprehensively review the literature and conducted this meta-analysis to show the role of NTx concentrations in the diagnosis of bone metastasis in lung cancer patients.

## Methods

### Literature search strategy and trial selection

Articles were retrieved from three English electronic databases of PubMed, Embase, Web of Science and two Chinese databases of China National Knowledge Infrastructure (CNKI) and Wanfang. The search period was from the start of each database up to December 2016. The search terms such as ‘lung cancer’, ‘lung neoplasm’, ‘lung malignancy’, ‘NTx’, ‘N-terminal type I collagen telopeptide’, ‘N-telopeptide’,‘bone metastasis’ were involved. The search strategy we used in Pubmed was: ((((N-telopeptide) OR N-terminal type I collagen telopeptide) OR NTx telopeptide) OR NTX) AND "Lung Neoplasms"[Mesh]. In addition to the databases searching, we had also reviewed the reference listed on included articles.

### Selection criteria

The inclusion criteria for this study were summarized as follows: (1) patients must be pathologically diagnosed with lung cancer; (2) Patients who were identified bone metastasis were confirmed by bone scintigraphy, emission computed tomography (ECT) or other imaging techniques.(3) studies must have a control group either patients without bone metastasis or healthy person; (4) studies must have reported sufficient quantitative data such as Mean±SD or data regarding sensitivity/specificity; (5) only the study with larger sample be included if multiple studies contained overlap data. Exclusion criteria: (1) non-human study; (2) studies without a control group; (3) studies without sufficient data to be extracted from; (4) non-original studies such as reviews, case reports.

### Data extraction

All data were extracted by two independent reviewers (Boxuan Liu and Lizhong Zeng) according to the above criteria. Once there was a discrepancy, a third reviewer (Xia Meng) would be asked to reach an agreement. The extracted data are summarized as follows: (1) general information, including the title, publication date, author, gender, age and histological classification; (2) design and implementation, including the type of design, test method, cut-off value, ethnics; (3) measurement indicators such as NTx level, the number of true positives, false positives, true negatives, and false negatives.

### Quality assessment

QUADAS and QUADAS-2were recommended assessment tools to evaluate the risk of bias and applicability of primary diagnostic accuracy studies[8][9]. Two reviewers (Jingyan Yuan and Yun Zhao) independently evaluated the included studies and discrepancies were resolved by consulting a third reviewer (Shuanying Yang).

### Statistical analysis

The weighted mean difference (WMD) and its 95% confidence intervals (CI) was used to measure NTx level from the data given in the included studies using fixed effect or random effect method judging by the heterogeneity which was evaluated by the Chi-square test and I^2^. Once I^2^<50% and *P*>0.1, we applied the fixed effect method, otherwise the random effect method was used. The overall effect of this meta-analysis was measured using Z-scores with a *P* <0.05. Sensitivity analysis was conducted in this study by omitting an included study each time to confirm the stability of the study. Begg’s and Egger’s test were applied to evaluate the publication bias. Further, we used a summary receiver operating characteristic (SROC) curve to illustrate the performance of sensitivity and specificity. Statistical analysis was conducted using Meta DiSc statistical software (Version 1.4, Madrid, Spain), RevMan 5.3 (Cochrane Collaboration) and Stata version 12.0 (TX, USA).

## Results

### Search results

**Fig 1:**
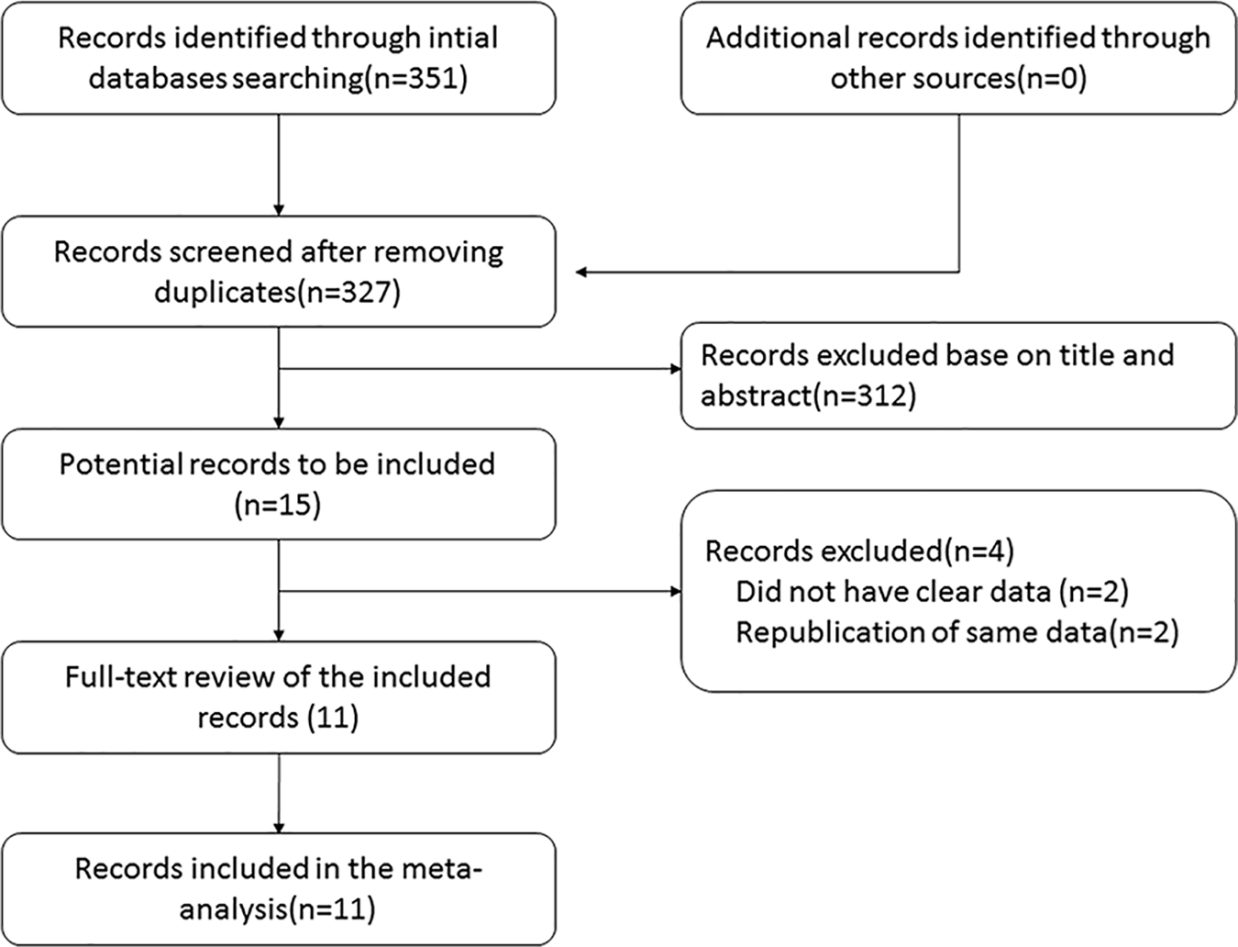
Flow chart for study search (PRISMA diagram).

According to our search strategy, we initially identified 351 studies, after removing the duplicates, 327 studies had a further text review of title and abstract. After a review of 327 studies, 15 studies were selected for a full-text review and 4 articles were excluded for the following reasons: insufficient data, republication of the same data. Thus, a total of 11 studies were eligible for this meta-analysis, A flowchart showing the study selection process is shown in Fig 1.

### Characteristics of the included studies

Of the 11 studies included in this meta-analysis, a total of 1108 patients were examined in which 438 had bone metastasis. The diagnostic definition of bone metastasis in each study was presented in S2 File. The sample size of included studies oscillated between 35 and 176. A total of 9 studies were conducted among Asian people while the rest two studies were carried out in Turkey and Italy. The histological classification of lung cancer mainly contained lung adenocarcinoma (LAC) (341patients), lung squamous cell carcinoma (LSCC) (142 patients) and small cell lung cancer(SCLC) (157 patients). More details of the studies are shown in Table 1.

**Table 1:** Baseline characteristics of included studies.

Author	Year	All cases(with BM/without BM)	Gender(M/F)(N)	Age(years)	Histology of lung cancer(N)				Tumor stage
					LAC	LSCC	SCLC	Others	
Lumachi F[10]	2011	35(16/19)	24/11	63(51–72)	NA	NA	0	0	I-IV
Tamiya M[11]	2013	176(73/103)	125/51	68(23–85)	109	53	NA	14	I-IV
Chung JH[12]	2005	151(33/118)	105/46	62(28–84)	NA	NA	NA	NA	I-IV
Tamiya M[13]	2013	150(50/100)	102/48	66(25–86)	56	16	20	8	I-IV
Bayrak SB[14]	2011	65(23/42)	65/0	64.07±8.7	NA	NA	15	0	I-IV
Izumi M [15]	2001	100(20/80)	67/33	64(41–82)	56	31	11	2	I-IV
Li WB [16]	2012	82(45/37)	NA	51.4±12.1	NA	NA	NA	NA	I-IV
ZhangSQ [17]	2011	106(61/45)	57/49	NA	40	26	31	9	I-IV
Chen SW [18]	2010	76(32/44)	55/21	58.3	0	0	76	0	I-IV
Sun H [19]	2013	100(53/47)	66/34	NA	80	16	4	0	I-IV
Xie WG [20]	2011	67(32/35)	NA	53.2	NA	NA	0	0	I-IV

**List of included studies.** NA unavailable; N cases; BM bone metastasis; LAC lung adenocarcinoma; LSCC lung squamous cell carcinoma; SCLC small cell lung cancer

### Quality assessment

All of the included studies had adopted enzyme-linked immunosorbent assay (ELISA) as the test method of NTx. Of the included studies, two studies used urine sample, eight studies tested serum sample and one study had measured both serum and urine sample. The cut-off values were ranged from 62.5nM BCE/Cr to 73.0nM BCE/Cr, 9nM BCE/L to 30nM BCE/L for urine and serum sample respectively. Two researchers (Y Zhao and JY Yuan) were assigned to evaluate all of the included studies independently based on the protocol of QUADAS and QUADAS-2. Their mean QUADAS score was 10.36 (ranged from 9 to 11), suggesting a generally good quality level. (Table 2, S3 File)

**Table 2:** Methodology and quality of included studies.

Author	Year	Research design	Country	Asians/Caucasians	Test method	Cut-off value	Specimen	QUADAS
Lumachi F[10]	2011	NA	Italy	0/35	ELISA	30nM BCE/L	serum	9
Tamiya M[11]	2013	Prospective	Japan	176/0	ELISA	22nM BCE/L	serum	10
Chung JH[12]	2005	Prospective	Korea	151/0	ELISA	73nM BCE/Cr	urine	10
Tamiya M[13]	2013	Prospective	Japan	150/0	ELISA	64nM BCE/Cr	urine	11
						22nM BCE/L	serum	
Bayrak SB[14]	2011	Prospective	Turkey	0/65	ELISA	25.69nM BCE/L	serum	10
Izumi M [15]	2001	Prospective	Japan	100/0	ELISA	62.5nM BCE/Cr	urine	11
Li WB [16]	2012	Retrospective	China	82/0	ELISA	NA	serum	11
Zhang SQ [17]	2011	Prospective	China	106/0	ELISA	9nM BCE/L	serum	11
Chen SW [18]	2010	NA	China	76/0	ELISA	NA	serum	11
Sun H [19]	2013	Proospective	China	100/0	ELISA	26.75nM BCE/L	serum	9
Xie WG [20]	2011	Proospective	China	67/0	ELISA	NA	serum	11

QUADAS quality assessment for studies of diagnostic accuracy (maximum score 14); ELISA enzyme-linked immunosorbent assays; NA unavailable

### Heterogeneity test

Chi-square value was used to determine heterogeneity among studies. With a larger value, there was more likely to have heterogeneity among studies. In our study, the Chi-square value of the studies with urine sample was 4.32 with 2° of freedom (P = 0.115). For studies with serum sample, the value was 40.82 with 7° of freedom (*P* < 0.01). The results indicated that there was more likely to have heterogeneity in serum sample studies. Thus, we subsequently reviewed the studies from various aspects and considered that specific histological type of lung cancer might contribute to this. However, all of the included studies had a good homogeneity of study design and clinical intention which was also an important aspect. Thus, we finally applied the random-effect model to perform this analysis.

### Comparison of NTx level between BM+ and BM- patients

**Fig 2:**
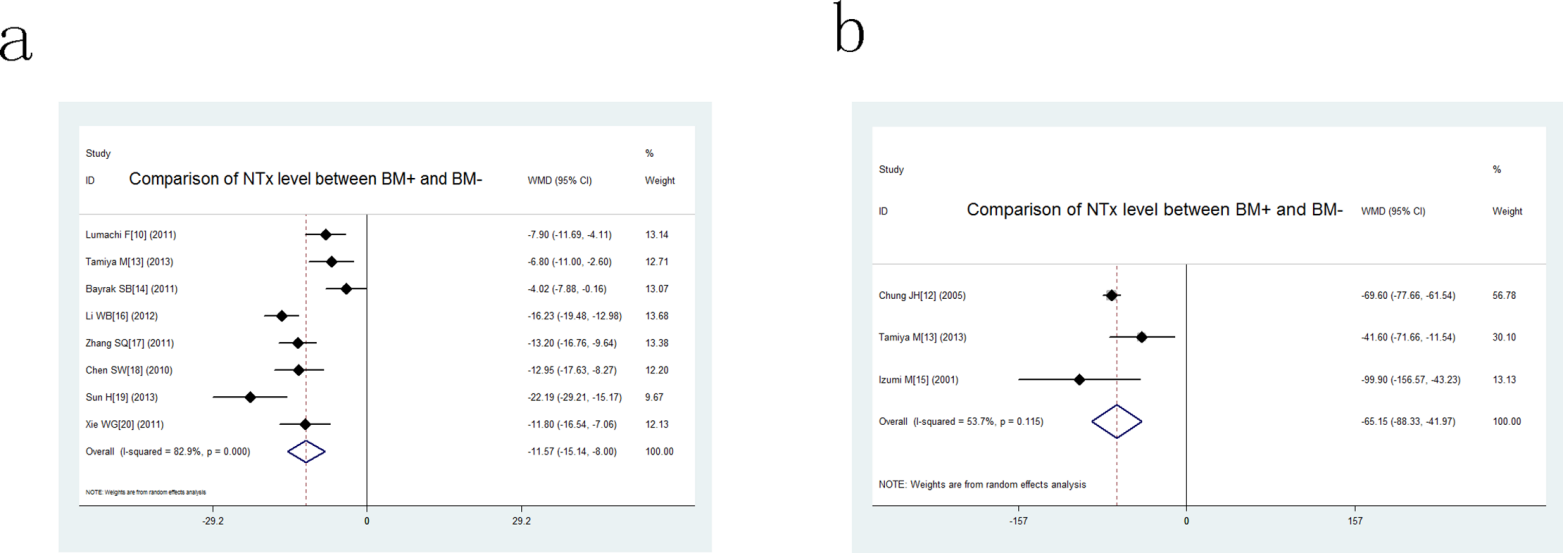
Forest plots for comparison of NTx level between BM+ and BM- in lung cancer patients. a: comparison of serum NTx level; b: comparison of urine NTx level.

Shown in Table 3, all the included studies compared the level of NTx in lung cancer with/without bone metastasis except one conducted by Tamiya which due to the lack of standard deviation. The weight of included studies ranged from -0.16% to -29.21%, -11.54% to -156.57% respectively for serum and urine sample. The pooled WMD was -11.57 and 95% confidence interval (CI) were -15.14 to -8.00 for serum sample, -65.15(95% CI -88.33 to -41.97) for urine sample (Fig 2), which indicated that patients with bone metastasis had a higher NTx level than those without bone metastasis regardless of the type of sample. Thus, the results indicated that higher NTx was a concomitant event of lung cancer patients suffered bone metastasis.

**Table 3:** Data extract of NTx level in studies.

Author	Concentration of NTx (Mean ± standard deviation)					Diagnostic test (2 × 2 table)			
	Without BM	With BM	LAC	LSCC	SCLC	TP	FP	FN	TN
Lumachi F[10]	25.6±3.1	33.5±7.2	NA	NA	NA	9	2	7	17
Tamiya M[11]	17.3	29.4	NA	NA	NA	45	14	18	89
Chung JH[12]	50.7±2.3	120.3±23.6	NA	NA	NA	24	19	9	99
Tamiya M[13]	51.6±26.8	93.2±105.1	NA	NA	NA	24	7	26	43
Bayrak SB[14]	18.67±6.85	22.69±7.98	NA	NA	NA	10	4	13	38
Izumi M[15]	47.2±29.9	147.1±129.3	NA	NA	NA	16	21	4	59
Li WB[16]	11.43±3.44	27.76±10.66	NA	NA	NA	NA	NA	NA	NA
Zhang SQ[17]	12.16±7.62	25.36±11.07	26.71±13.13	25.21±12.60	24.93±12.76	55	3	6	38
Chen SW[18]	13.02±8.76	25.97±11.25	NA	NA	25.97±11.25	NA	NA	NA	NA
Sun H[19]	23.99±9.05	46.18±24.22	NA	NA	NA	40	11	13	36
Xie WG[20]	13.21±7.59	25.01±11.67	NA	NA	NA	NA	NA	NA	NA

NA, unavailable; true positive; LAC lung adenocarcinoma; LSCC lung squamous cell carcinoma; SCLC small cell lung cancer; TP true positive FP false positive; FN false negative; TN true negative

### Analysis of sensitivity and publication bias

**Fig 3:**
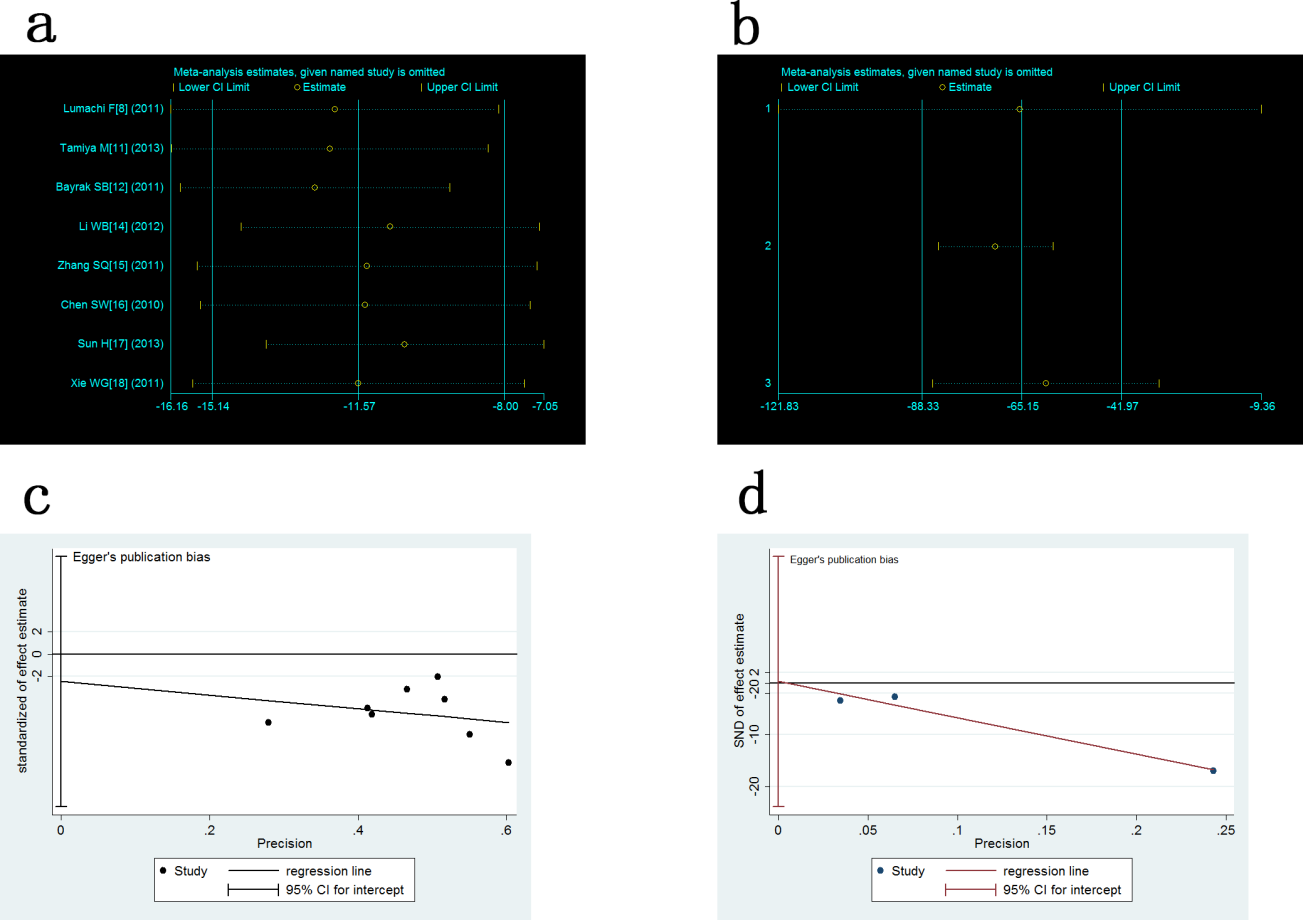
Analysis of sensitivity and publication bias. a: sensitivity analysis for comparison of serum NTx level between BM+ and BM- in lung cancer patients; b: sensitivity analysis for comparison of urine NTx level between BM+ and BM- in lung cancer patients; c: Egger test of serum NTx; d: Egger test of urine NTx.

The sensitivity analysis showed that the exclusion of one study each time did not significantly modify the estimators and alter the final outcome, with a pooled WMD oscillating between -16.16 to -7.05, -121.83 to -9.36 respectively for serum and urine sample (Fig 3A and 3B). We applied the Egger test and Begg’s Test to evaluate whether there was a publication bias or not. The results showed that t value of the Egger test was -0.53 (P > |t| = 0.612) and 0.15 (P > |t| = 0.904), z value of Begg’s Test was 0.25 (Pr > |Z| = 0.805) and -0.52 (Pr > |Z| = 0.602) respectively in serum and urine studies. With such values, no publication bias was observed (Fig 3C and 3D).

### Sensitivity and specificity of NTx for distinguishing bone metastasis

**Fig 4:**
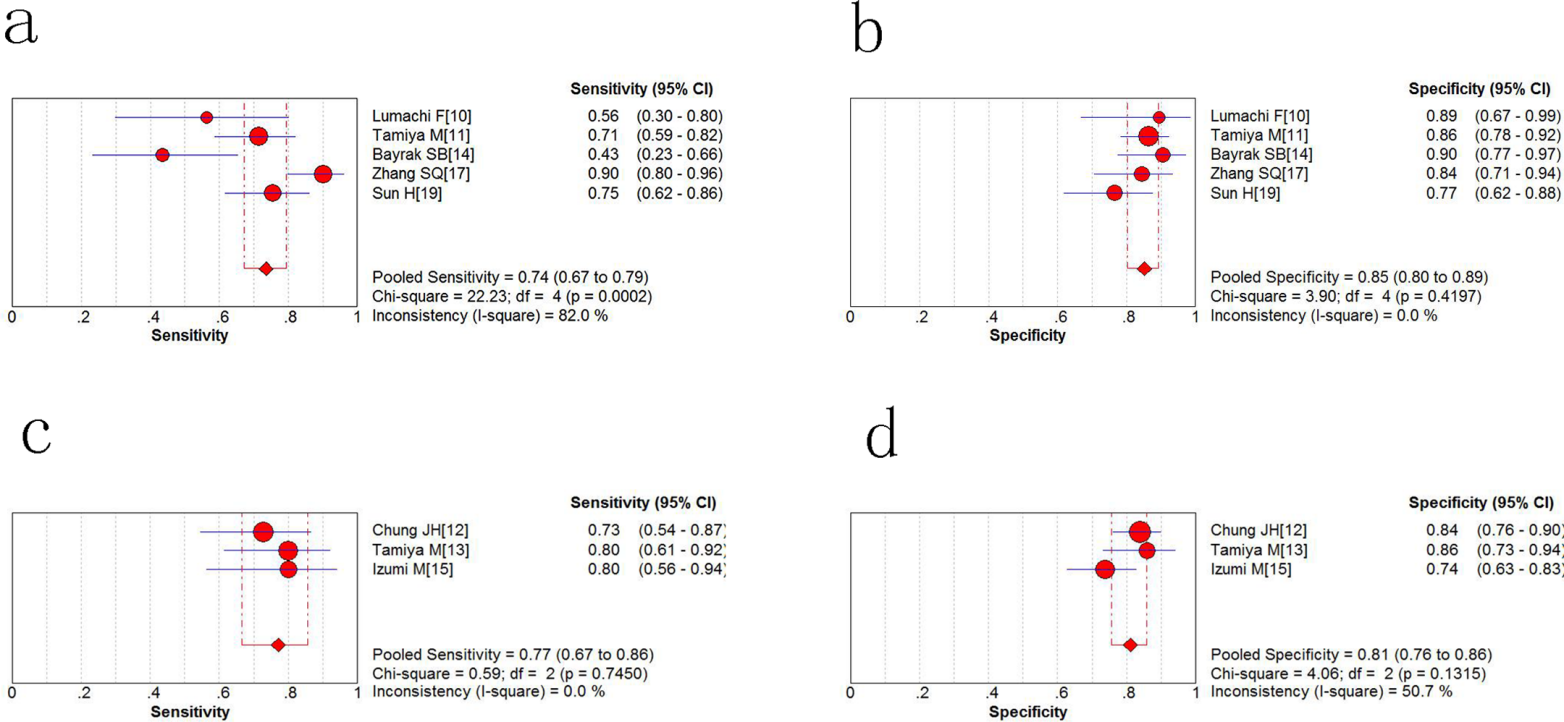
Sensitivity and specificity of NTx for the diagnosis of lung cancer. a Pooled sensitivity for serum NTx; b pooled specificity for serum NTx; c Pooled sensitivity for urine NTx; d pooled specificity for urine NTx.

As shown in the forest plot (Fig 4A), the pooled sensitivity of serum NTx (sNTx) in five included studies was 0.74 (95% confidence interval = 0.67 to 0.79) and the pooled specificity (Fig 4B) of sNTx was 0.85 (95% CI, 0.80 to 0.89). As for urine NTx(uNTx), the pooled sensitivity was 0.77(95% CI = 0.67 to 0.86) and specificity was 0.81(95% CI = 0.76 to 0.86). (Fig 4C and 4D) Both sNTx and uNTx had a higher specificity over sensitivity in discerning bone metastasis among lung cancer patients.

### Diagnostic accuracy of NTx for discerning bone metastasis

**Fig 5:**
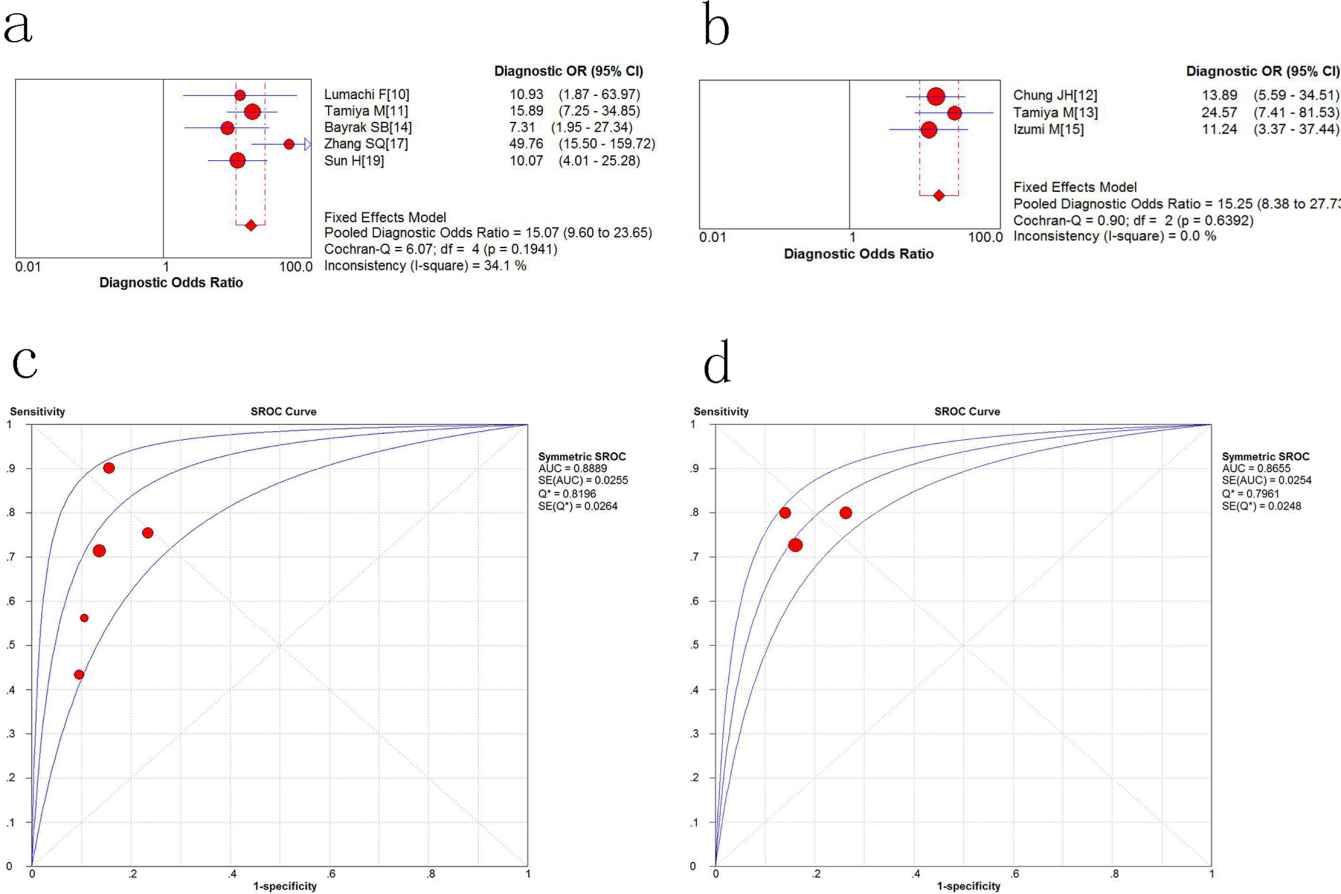
Diagnostic accuracy of NTx to bone metastasis. a The overall Diagnostic Odds Ratio (DOR) of serum NTx; b the overall DOR of urine NTx; c the SROC(summary receiver operating characteristic) curve for serum NTx; d he SROC curve for urine NTx.

The overall diagnostic odds ratio (DOR) is a measure of the effectiveness of a diagnostic test. In our study, the sNTx is 15.07 (9.6 to 23.65, P = 0.1941) and for uNTx the DOR is 15.25 (8.38 to 27.73, P = 0.6392). (Fig 5A and 5B). With a high DOR value, it is indicative that NTx had a better test performance. Fig 5C and 5D summarize the serum and urine NTx performance by using the SROC (summary receiver operating characteristic) curve, and the balanced point for sensitivity and specificity (the Q-value) was 0.8196 and 0.7961 respectively. The area under the curve (AUC) was 0.8889 and 0.8655, indicating that the overall accuracy was impressive both in serum and urine sample.

## Discussion

So far, lung cancer has become the leading cause of cancer-related deaths worldwide and the 5-year survival rate is only slightly above 10%[1]. A previous study shows that in the clinical course of lung cancer, a third of all patients will suffer from bone metastasis and the SREs significantly affects clinical outcome with a median survival time of 4.1 months[21]. NTx, a biochemical marker of bone metabolism, is considered to be elevated during bone metastasis because it reflects the ongoing rate of bone osteolysis. Thus the elevation of NTx has been further discussed as a diagnostic biomarker in solid tumors [22–24]. A previous meta-analysis[7] has demonstrated an elevation of NTx in bone metastasis patients, however, it fails to calculate the sensitivity and specificity of NTx in the diagnosis of bone metastasis which would be an important data for further clinical trial and application. Besides, the patients included have different kinds of tumor which may result in bias when applied in lung cancer patients. Moreover, serum and urine are the two sources of specimen in detecting NTx but the study has failed to evaluate urine NTx and its correlation with serum NTx in terms of efficacy. Therefore, we review the literature so as to make a more comprehensive analysis on the role of NTx as a diagnostic biomarker.

In this meta-analysis, after reviewing the relevant studies comparing NTx level between BM+ and BM- lung cancer patients, we found a higher level of NTx occurred in BM+ patients regardless of serum or urine sample. This indicated that the elevation of NTx positively correlated with the occurrence of bone metastasis and it could be a potential biomarker. We also noticed that there was a strong heterogeneity exist between included studies and subsequent subgroup analysis based on the type of specimen did not diminish such heterogeneity. However, we found that among the included studies there was a good clinical homogeneity which we believed to be another important factor. For example, no biases of age, gender were observed in these studies. What is more, the test method in each of the studies were unanimously ELISA. Moreover, most of the studies had a moderate to higher quality assessed by the QUADAS system. Subsequent sensitivity analysis further showed that the exclusion of studies on an individual basis did not substantially change the overall result of this meta-analysis. Bias evaluation in our study also suggested there was no significant publication bias exist. Together, we believe higher NTx value is a potential indicator of bone metastasis in lung cancer patients.

These days, bone metabolic markers have been extensively studied and have shown its ability as a useful clinical tool in detecting bone metastasis. Based on their origin, bone metabolic markers can be divided into markers of formation and resorption. Bone-specific alkaline phosphatase (B-ALP), C-terminal or N-terminal propeptides of type I procollagen (PICP; PINP) are bone formation markers while tartrate-resistant acid phosphatase isoform 5b (TRACP 5b), type I collagen carboxyterminal telopeptide (ICTP) and NTx are the main bone resorption markers.

It is no doubt that good sensitivity and specificity are essential for a diagnostic marker. In this meta-analysis we found that the increase of NTx both in serum and urine has a higher specificity (sNTx: 0.85; 95% CI: 0.80–0.89; uNTx: 0.81; 95% CI = 0.76 to 0.86) over sensitivity (sNTx: 0.74; 95%CI: 0.67 to 0.79; uNTx: 0.77; 95% CI = 0.67 to 0.86) which suggested that NTx has a better role for detecting patients who have not been affected by bone metastasis. Thus, a preliminary test of NTx can be a complementary tool to current methodology and make it more accurate and cost-effective. However, a normal level of NTx does not completely rule out the possibility of bone metastasis. The DOR is a measurement of the effectiveness of a diagnostic test and synthesizes sensitivity and specificity into a quantitative data. People believe that a higher DOR values indicate a better test performance. In our analysis, the pooled DOR was 15.07 and 15.25 for sNTx and uNTx respectively, supporting that the NTx test could be advantageous in the diagnostic test of bone metastasis. The SROC curve is a graphical plot that can illustrate the performance of a diagnostic test. Our analysis showed that the AUC of NTx was 0.8889 and 0.8655 in serum and urine sample, which showed that the NTx has good value in terms of the discerning bone metastasis of lung cancer. From the present data, both serum and urine sample show a similar diagnostic efficacy, however, the serum sample may be a better option for it is more flexible regarding specimen collection. Judging from the research, we believe that every patient with lung cancer should undergo the test of NTx.For one thing, it is a more convenient and cost-effective way in discerning bone metastasis, for another thing, with an elevated NTx the current methodology will be more effective and accurate to draw a final diagnosis.

When compared with other markers, NTx shows a better diagnostic accuracy. A meta-analysis[25] on B-ALP shows the sensitivity (74%) is similar to that of NTx while the specificity (80%) is inferior to that of NTx. In another study conducted by Tang *et al*[26], the sensitivity values decrease in the order ICTP (63.1%), B-ALP (63.1%), and TRACP 5b (58.5%) and the corresponding specificity values are 90.4%, 77%, and 80.4% respectively. Even if ICTP has a better specificity than NTx, the highest AUC (0.835) is inferior to that of NTx indicating NTx has a better overall diagnostic efficiency. Finally, Ebert *et al* [27]concludes the area under SROC curves for B-ALP, ICTP and PINP are 0.764, 0.699, and 0.742. Therefore, NTx so far has the highest diagnostic efficiency compared with other markers in detecting bone metastasis in lung cancer.

The limitations of this study are as follows: first, the current results were based on relatively small number of studies and lacks a large sample size; second, significantly heterogeneity was observed among studies that cannot be fully eliminated due to lack of a more detailed data; third, the cutoff value for positive of NTx varied across included studies and might interfere the outcome of current meta-analysis. In the future, it is very crucial to compare the NTx status in different histology classification of lung cancer suffered from bone metastasis in multiple clinical centers with large samples. Although some deficiencies existed, the study still drew a conclusion that the NTx could be beneficial in the diagnosis of bone metastasis in lung cancer.

### Conclusion

In summary, patients with bone metastasis have a high level of sNTx and uNTx than those without bone metastasis indicating increased NTx positively correlates with the occurrence of bone metastasis. Furthermore, both uNTx and sNTx have a relatively high sensitivity and specificity and can be a biomarker in the diagnosis of bone metastasis in lung cancer patients.

## Supporting information

S1 File PRISMA 2009 checklist. (DOC)

S2 File Diagnostic definition of bone metastasis in each studies. (DOCX)

S3 File Quality assessment of studies included in the meta-analysis using QUADAS and QUADAS-2. (DOCX)

## References

[1] Ferlay J, Soerjomataram I, Dikshit R, Eser S, Mathers C, Rebelo M, et al Cancer incidence and mortality worldwide: sources, methods and major patterns in GLOBOCAN 2012. Int J Cancer. 2015;136(5):E359–86. doi: 10.1002/ijc.29210 . 25220842 10.1002/ijc.29210

[2] Coleman RE. Skeletal complications of malignancy. Cancer. 1997;80(8 Suppl):1588–94. . 9362426 10.1002/(sici)1097-0142(19971015)80:8+<1588::aid-cncr9>3.3.co;2-z

[3] Coleman RE. Clinical features of metastatic bone disease and risk of skeletal morbidity. Clinical cancer research: an official journal of the American Association for Cancer Research. 2006;12(20 Pt 2):6243s–9s. doi: 10.1158/1078-0432.CCR-06-0931 . 17062708 10.1158/1078-0432.CCR-06-0931

[4] Silvestri GA, Littenberg B, Colice GL. The clinical evaluation for detecting metastatic lung cancer. A meta-analysis. American journal of respiratory and critical care medicine. 1995;152(1):225–30. doi: 10.1164/ajrccm.152.1.7599828 . 7599828 10.1164/ajrccm.152.1.7599828

[5] Simon LS, Krane SM, Wortman PD, Krane IM, Kovitz KL. Serum levels of type I and III procollagen fragments in Paget's disease of bone. The Journal of clinical endocrinology and metabolism. 1984;58(1):110–20. doi: 10.1210/jcem-58-1-110 . 6227628 10.1210/jcem-58-1-110

[6] Pectasides D, Nikolaou M, Farmakis D, Kanakis I, Gaglia A, Kountourakis P, et al Clinical value of bone remodelling markers in patients with bone metastases treated with zoledronic acid. Anticancer research. 2005;25(2B):1457–63. . 15865105

[7] Zhang Y, Yi M, Cao J, Hou C, Zhou Y, Zhong Y. Serum cross-linked N-telopeptide of type I collagen for the diagnosis of bone metastases from solid tumours in the Chinese population: Meta-analysis. J Int Med Res. 2016;44(2):192–200. doi: 10.1177/0300060515600187 . 26857861 10.1177/0300060515600187 PMC5580071

[8] Whiting PF, Rutjes AW, Westwood ME, Mallett S, Deeks JJ, Reitsma JB, et al QUADAS-2: a revised tool for the quality assessment of diagnostic accuracy studies. Ann Intern Med. 2011;155(8):529–36. doi: 10.7326/0003-4819-155-8-201110180-00009 . 22007046 10.7326/0003-4819-155-8-201110180-00009

[9] Whiting P, Rutjes AW, Reitsma JB, Bossuyt PM, Kleijnen J. The development of QUADAS: a tool for the quality assessment of studies of diagnostic accuracy included in systematic reviews. BMC medical research methodology. 2003;3:25 doi: 10.1186/1471-2288-3-25 ; PubMed Central PMCID: PMC305345. 14606960 10.1186/1471-2288-3-25 PMC305345

[10] Lumachi F, Marino F, Fanti G, Chiara GB, Basso SM. Serum N-telopeptide of type I collagen and bone alkaline phosphatase and their relationship in patients with non-small cell lung carcinoma and bone metastases. Preliminary results. Anticancer research. 2011;31(11):3879–81. . 22110213

[11] Tamiya M, Kobayashi M, Morimura O, Yasue T, Nakasuji T, Satomu M, et al Clinical significance of the serum crosslinked N-telopeptide of type I collagen as a prognostic marker for non-small-cell lung cancer. Clinical lung cancer. 2013;14(1):50–4. doi: 10.1016/j.cllc.2012.03.012 . 22609050 10.1016/j.cllc.2012.03.012

[12] Chung JH, Park MS, Kim YS, Chang J, Kim JH, Kim SK, et al Usefulness of bone metabolic markers in the diagnosis of bone metastasis from lung cancer. Yonsei medical journal. 2005;46(3):388–93. doi: 10.3349/ymj.2005.46.3.388 ; PubMed Central PMCID: PMC2815816. 15988811 10.3349/ymj.2005.46.3.388 PMC2815816

[13] Tamiya M, Tokunaga S, Okada H, Suzuki H, Kobayashi M, Sasada S, et al Prospective study of urinary and serum cross-linked N-telopeptide of type I collagen (NTx) for diagnosis of bone metastasis in patients with lung cancer. Clinical lung cancer. 2013;14(4):364–9. doi: 10.1016/j.cllc.2012.11.006 . 23276824 10.1016/j.cllc.2012.11.006

[14] Bayrak SB, Ceylan E, Serter M, Karadag F, Demir E, Cildag O. The clinical importance of bone metabolic markers in detecting bone metastasis of lung cancer. International journal of clinical oncology. 2012;17(2):112–8. doi: 10.1007/s10147-011-0266-7 . 21691728 10.1007/s10147-011-0266-7

[15] Izumi M, Nakanishi Y, Takayama K, Kimotsuki K, Inoue K, Wataya H, et al Diagnostic value of bone-turnover metabolites in the diagnosis of bone metastases in patients with lung carcinoma. Cancer. 2001;91(8):1487–93. . 11301396 10.1002/1097-0142(20010415)91:8<1487::aid-cncr1156>3.0.co;2-2

[16] Li WB, Luo HJ, Wang P, et al Diagnostic value of ECT combined CYFRA21-1, NTx, BSP in lung cancer with bone metastasis. Journal of Hubei Medical University 2013, 32:111–114 [in Chinese]

[17] Zhang SQ, Chen DB, Wang BQ, et al Relationships between the levels of serum NTx, ICTP, BAP and bone metastasis in patients with lung cancer. Chinese Clinical Oncology 2011; 16: 534–537 [in Chinese]

[18] Chen SW, Xia B and Xu J. Clinical significance of serum NTx and BSP in non-smallcell lung cancer with bone metastases. Chinese General Practice 2010; 13: 1771–1772 [in Chinese]

[19] Sun H, Chen XX, Zhao YM, et al NTx in diagnosis and evaluation of efficacy in lung cancer with bone metastases. Chinese Journal of Thoracic and Cardiovascular Surgery 2013; 29: 172–173 [in Chinese]

[20] Xie WG, Wang L, Xie Z, et al Value of serum NTx and BSP in diagnosing non-small cell lung cancer with bone metastasis. Journal of Clinical Pulmonary Medicine 2011; 16: 1898–1899 [in Chinese]

[21] Delea T, Langer C, McKiernan J, Liss M, Edelsberg J, Brandman J, et al The cost of treatment of skeletal-related events in patients with bone metastases from lung cancer. Oncology. 2004;67(5–6):390–6. doi: 10.1159/000082923 . 15713995 10.1159/000082923

[22] Lipton A, Cook R, Saad F, Major P, Garnero P, Terpos E, et al Normalization of bone markers is associated with improved survival in patients with bone metastases from solid tumors and elevated bone resorption receiving zoledronic acid. Cancer. 2008;113(1):193–201. doi: 10.1002/cncr.23529 . 18459173 10.1002/cncr.23529

[23] Brown JE, Cook RJ, Major P, Lipton A, Saad F, Smith M, et al Bone turnover markers as predictors of skeletal complications in prostate cancer, lung cancer, and other solid tumors. Journal of the National Cancer Institute. 2005;97(1):59–69. doi: 10.1093/jnci/dji002 . 15632381 10.1093/jnci/dji002

[24] Brown JE, Thomson CS, Ellis SP, Gutcher SA, Purohit OP, Coleman RE. Bone resorption predicts for skeletal complications in metastatic bone disease. British journal of cancer. 2003;89(11):2031–7. doi: 10.1038/sj.bjc.6601437 ; PubMed Central PMCID: PMC2376859. 14647134 10.1038/sj.bjc.6601437 PMC2376859

[25] Du WX, Duan SF, Chen JJ, Huang JF, Yin LM, Tong PJ. Serum bone-specific alkaline phosphatase as a biomarker for osseous metastases in patients with malignant carcinomas: a systematic review and meta-analysis. J Cancer Res Ther. 2014;10 Suppl:C140–3. doi: 10.4103/0973-1482.145842 . 25450272 10.4103/0973-1482.145842

[26] Tang C, Liu Y, Qin H, Li X, Guo W, Li J, et al Clinical significance of serum BAP, TRACP 5b and ICTP as bone metabolic markers for bone metastasis screening in lung cancer patients. Clin Chim Acta. 2013;426:102–7. doi: 10.1016/j.cca.2013.09.011 . 24055775 10.1016/j.cca.2013.09.011

[27] Ebert W, Muley T, Herb KP, Schmidt-Gayk H. Comparison of bone scintigraphy with bone markers in the diagnosis of bone metastasis in lung carcinoma patients. Anticancer Res. 2004;24(5B):3193–201. . 15510610

