# Supplementary material for: Elevated N-telopeptide as a potential diagnostic marker for bone metastasis in lung cancer: A meta-analysis
Source: PLoS One. 2017 Nov 28;12(11):e0187860. doi: 10.1371/journal.pone.0187860 (PMC5705147; doi:10.1371/journal.pone.0187860)
Supplement: S2 File — (DOCX) [file pone.0187860.s002.docx]

**Supplement 2.Diagnostic definition of bone metastasis in each studies**

| Study ID | Diagnostic definition of bone metastasis |
| --- | --- |
| Lumachi F[[10](#_ENREF_8)] | 1. pathologically confirmed lung cancer; 2. osteolytic lesion confirmed by bone scintigraphy, CT or MRI |
| Tamiya M[[11](#_ENREF_9)] | 1. pathologically confirmed lung cancer; 2. concentration foci confirmed assessed by bone scintigraphy; 3. CT, MRI or PET-CT were used for a more detailed evaluation if could not be confirmed. |
| Chung JH[[12](#_ENREF_10)] | 1. pathologically confirmed lung cancer; 2. bone scintigraphy assessed by two radiologists; 3. If discrepancy occurred, CT, MRI or PET-CT were used for a more detailed evaluation. |
| Tamiya M[[13](#_ENREF_11)] | 1. pathologically confirmed lung cancer; 2. concentration foci confirmed by bone scintigraphy; 3. CT, MRI or PET-CT were used for a more detailed evaluation if could not be confirmed. |
| Bayrak SB[[14](#_ENREF_12)] | Not mentioned |
| Izumi M [[15](#_ENREF_13)] | 1. pathologically confirmed lung cancer; 2. osteolytic lesion confirmed by bone scintigraphy, CT or MRI; 3.clinical findings such as: pathological fracture, [hypercalcemia](javascript:void(0);) or SREs |
| Li WB [16] | 1. pathologically confirmed lung cancer; 2. osteolytic lesion confirmed by bone scintigraphy or multiple concentration foci（＞2） confirmed by bone scintigraphy |
| Zhang SQ [17] | 1. pathologically confirmed lung cancer; 2. abnormal concentration foci assessed by bone scintigraphy; 3. osteolytic lesion confirmed by CT or MRI |
| Chen SW [18] | 1. pathologically confirmed lung cancer; 2. abnormal concentration foci assessed by bone scintigraphy and confirmed by CT or MRI; 3. abnormal concentration foci assessed by bone scintigraphy with clinical symptoms; 4. confirmed by PET-CT. |
| Sun H [19] | Not mentioned |
| Xie WG [20] | 1. pathologically confirmed lung cancer; 2. abnormal concentration foci assessed by bone scintigraphy and further confirmed by CT or MRI or with specific clinical symptoms such as pathological fracture, [hypercalcemia](javascript:void(0);) or SREs. |
