# Supplementary material for: Elevated N-telopeptide as a potential diagnostic marker for bone metastasis in lung cancer: A meta-analysis
Source: PLoS One. 2017 Nov 28;12(11):e0187860. doi: 10.1371/journal.pone.0187860 (PMC5705147; doi:10.1371/journal.pone.0187860)

**Supplement 3.Quality assessment of studies included in the meta-analysis using QUADAS score**

| **Study ID** | Lumachi F[[10](#_ENREF_8)] | Tamiya M[[11](#_ENREF_8)] | Chung JH[[12](#_ENREF_10)] | Tamiya M[[13](#_ENREF_8)] | Bayrak SB[[14](#_ENREF_12)] | Izumi M [[15](#_ENREF_13)] | Li WB[16] | Zhang SQ [17] | Chen SW [18] | Sun H [19] | Xie WG [20] |
| --- | --- | --- | --- | --- | --- | --- | --- | --- | --- | --- | --- |
| Was the spectrum of patients representative of the patients who will receive the test in practice? | Yes | Yes | Yes | Yes | Yes | Yes | Yes | Yes | Yes | Yes | Yes |
| Were selection criteria clearly described? | Unclear | Yes | Yes | Yes | Unclear | Yes | Yes | Yes | Yes | Unclear | Yes |
| Is the reference standard likely to correctly classify the target condition? | Yes | Yes | Yes | Yes | Yes | Yes | Yes | Yes | Yes | Yes | Yes |
| Is the time period between reference standard and index test short enough to be reasonably sure that the target condition did not change between the two tests? | Yes | Yes | Yes | Yes | Yes | Yes | Yes | Yes | Yes | Yes | Yes |
| Did the whole sample or a random selection of the sample, receive verification using a reference standard of diagnosis? | Yes | Yes | Yes | Yes | Yes | Yes | Yes | Yes | Yes | Yes | Yes |
| Did patients receive the same reference standard regardless of the index test result? | Yes | Yes | Yes | Yes | Yes | Yes | Yes | Yes | Yes | Yes | Yes |
| Was the reference standard independent of the index test (i.e. the index test did not form part of the reference standard)? | Yes | Yes | Yes | Yes | Yes | Yes | Yes | Yes | Yes | Yes | Yes |
| Was the execution of the index test described in sufficient detail to permit replication of the test? | Unclear | No | Yes | Yes | Yes | Yes | Yes | Yes | Yes | Yes | Yes |
| Was the execution of the reference standard described in sufficient detail to permit its replication? | No | Unclear | Unclear | Unclear | No | Unclear | Yes | Unclear | Yes | No | Yes |
| Were the reference standard results interpreted without knowledge of the results of the index test? | Yes | Yes | Yes | Yes | Yes | Yes | Yes | Yes | Yes | Yes | Yes |
| Were the index test results interpreted without knowledge of the results of the reference standard? | No | No | No | No | No | No | No | No | No | No | No |
| Were the same clinical data available when test results were interpreted as would be available when the test is used in practice? | Yes | Yes | No | Yes | Yes | Yes | Unclear | Yes | Unclear | Unclear | Unclear |
| Were uninterpretable/ intermediate test results reported? | Unclear | Unclear | Unclear | Unclear | Unclear | Unclear | Unclear | Unclear | Unclear | Unclear | Unclear |
| Were withdrawals from the study explained? | Yes | Yes | Yes | Yes | Yes | Yes | Yes | Yes | Yes | Yes | Yes |
| **Total** | 9 | 10 | 10 | 11 | 10 | 11 | 11 | 11 | 11 | 9 | 11 |


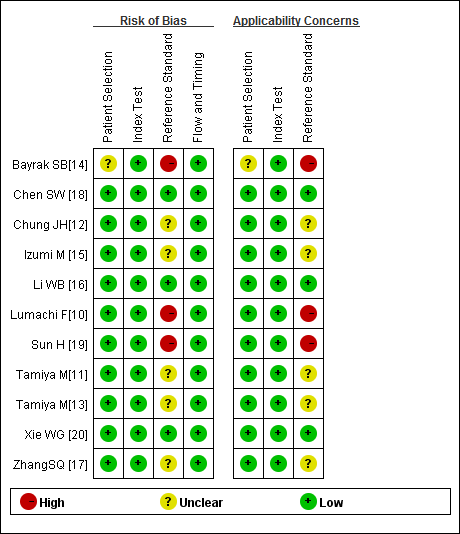

Supplement: S3 File — (DOCX) [file pone.0187860.s003.docx]
